# A comparative study of autogenous, allograft and artificial bone substitutes on bone regeneration and immunotoxicity in rat femur defect model

**DOI:** 10.1093/rb/rbaa040

**Published:** 2020-09-30

**Authors:** Wen Zou, Xing Li, Na Li, Tianwei Guo, Yongfu Cai, Xiaoqin Yang, Jie Liang, Yong Sun, Yujiang Fan

**Affiliations:** 1 National Engineering Research Center for Biomaterials, Sichuan University, 29 Wangjiang Road, Chengdu 610064, Sichuan, China; 2 Sichuan Testing Centre for Biomaterials and Medical Devices, 29 Wangjiang Road, Chengdu 610064, Sichuan, China

**Keywords:** autogenous bone, allogenic bone, artificial bone, bone defect repair, osteogenesis, immunotoxicity

## Abstract

Repair and reconstruction of large bone defect were often difficult, and bone substitute materials, including autogenous bone, allogenic bone and artificial bone, were common treatment strategies. The key to elucidate the clinical effect of these bone repair materials was to study their osteogenic capacity and immunotoxicological compatibility. In this paper, the mechanical properties, micro-CT imaging analysis, digital image analysis and histological slice analysis of the three bone grafts were investigated and compared after different time points of implantation in rat femur defect model. Autogenous bone and biphasic calcium phosphate particular artificial bone containing 61.4% HA and 38.6% β-tricalcium phosphate with 61.64% porosity and 0.8617 ± 0.0068 g/cm^3^ density (*d* ≤ 2 mm) had similar and strong bone repair ability, but autogenous bone implant materials caused greater secondary damage to experimental animals; allogenic bone exhibited poor bone defect repair ability. At the early stage of implantation, the immunological indexes such as Immunoglobulin G, Immunoglobulin M concentration and CD4 cells’ population of allogenic bone significantly increased in compared with those of autologous bone and artificial bone. Although the repair process of artificial bone was relatively inefficient than autologous bone graft, the low immunotoxicological indexes and acceptable therapeutic effects endowed it as an excellent alternative material to solve the problems with insufficient source and secondary trauma of autogenous bone.

## Introduction

Bone defect could easily lead to non-union and loss of bone functions, which greatly affects the life quality of patients. Orthopedics is usually used to stimulate fracture healing and reconstruct the lost native anatomy [1, 2]. For large bone defects, it was often difficult for self-healing. Thus, implantation of bone substitute materials, including autogenous bone, allogenic bone, heterogenous bone, and artificial bone substitutes, is often necessary in the main treatment method [3, 4]. Autogenous bone transplantation was considered as gold standard for clinical treatment of bone defect and non-union [5], since autologous bone contained many growth factors that were necessary for bone formation, such as bone morphogenetic protein, fibroblast growth factor, transfer growth factor and osteoinductive factors, which could stimulate the growth of bone and microvascular [6]. However, clinical practicability was an important factor for limiting autogenous bone transplantation [7]. In the process of bone removal, transplantation increased the surgical incision and the risk of trauma and complications [8]. It also destroyed the normal skeletal structure of donor site and affected its functional stability, which lead to donor area complications such as bleeding, infection and pain [9, 10]. 

Allogenic bone substitutes had relatively extensive sources and were easy to process and store. There was no risk of donor site injury as autogenous bone transplantation. Allogenic bone had an open, porous and reticulated physical structure similar with that of autogenous bone, which was conducive to the vascularization of bone after implantation. It is reported that the healing time of fracture implanted with allogenic bone substitute was close to that of autogenous bone [11]. Based on the above characteristics, the clinical application of allogenic bone was expanded [9, 12–15]. However, allogenic bone substitutes were easy to be absorbed and cause bone fracture [16]. Meanwhile, the immunogenicity risk was also another potential problem, which caused the failure of bone repair [17–19].

Artificial bone grafts such as hydroxyapatite (HAp), β-tricalciumphosphate (β-TCP) and bioactive glasses represented promising alternatives because they did not have some of the drawbacks mentioned above. After the implantation of artificial bone grafts *in vivo*, it could interact with body liquid and support the formation of new bone tissue. On the other hand, artificial bone grafts might adsorb and desorb functional proteins, resulting in changes in protein conformation [5, 11, 20–25]. This effect might change the immunological function *in vivo* and cause immunotoxicity [26]. Besides, the poor mechanical properties, especially brittleness restricted its clinical applications [27, 28]. Some literatures reported that biocompatible bone scaffolds with good mechanical properties could be generated by introduction of bioactive particles or nanosheets into polymer scaffold [29, 30].

Calcium phosphate bioceramics are important type of artificial bone materials. Porous biphasic calcium phosphate bioceramic had osteoinducing property, and could promote the regeneration of defected bone, enhance the bone integration and promote the new bone formation [31–34]. It had been clinically applied and showed particular advantage, comparing with other kinds of artificial bone substitute materials. However, there was no comprehensive study on its repair ability comparing with autogenous and allograft bone substitutes, from the aspect of bone formation. In this work, a comparative study on the effect of autogenous bone, allogenic bone and a biphasic calcium phosphate artificial bone substitute in repairing bone defects and immunotoxicology was carried out. Through the comparative study, the corresponding relationship between different bone grafts and bone repair effect were studied, such as biocompatibility, mechanical strength, osteoconductive properties, immunotoxicity, etc. [35]. This work would seek the best balance among the physicochemical and biological properties as well as immunotoxicity of bone grafts to support the selection of bone repair materials.

## Materials and methods

### Materials

Biphasic calcium phosphate ceramics particles with 61.64% porosity and 0.8617 ± 0.0068 g/cm^3^ density (*d* ≤ 2mm) were purchased from National Engineering Research Center for Biomaterials (Sichuan University, China). Enzyme-linked immunosorbent assay (ELISA) kit for Immunoglobulin G (IgG) and ELISA kit for Immunoglobulin M (IgM) were purchased from Cloud-Clone Corp (USA). Fluorescein isothiocyanate (FITC) mouse Anti-Rat CD4 and PE mouse Anti-Rat CD8a were purchased from BioLegend (USA). Roswell Park Memorial Institute 1640 (RPMI 1640) and fetal bovine serum (FCS) were purchased from HYCLONE (USA).

Experimental rats were purchased from Chengdu Dashuo Experimental Animal Co., Ltd (China). All animal studies were approved by the Sichuan University Medical Ethics Committee. All animal procedures were performed in accordance with the guidelines for care and use of laboratory animals of Sichuan University.

### Preparation of bone substitute materials

Autogenous bone was collected from anesthetized animals. A longitudinal on the skin over the iliac bone area was created, then the soft tissue attached to the iliac bone was separated. The iliac bone tissue was separated and removed using bone scissors and hemostatic forceps, and was used for transplantation directly. Allogenic bone substitute was prepared according to a previously published literature by destroying or even removing rat antigens through freezing in a refrigerator at −80°C for 2 weeks [36]. The artificial bone substitute material was the calcium phosphate ceramics as above described.

### Characterization of bone materials

The surface morphology and the crystal structure of materials were analyzed by scanning electron microscope (SEM, JSM-5900LV, JEOL) and X-ray diffraction (XRD) (X’Pert Pro MPD DY129, Nalytical). The SEM samples were prepared. For XRD, scanning range was from 20° to 80° with step size of 0.04° and step time of 1 s. For microscopic observation of autogenous bone and allogenic bone substitutes, samples were prepared by gradient dehydration in series concentration of ethanol and critical-point drying.

### *In vivo* femur bone implantation of bone substitutes

For *in vivo* study, murine femur defect model was chosen to estimate bone formation. The experimental animals were divided into three groups: autogenous bone group, artificial bone group and allogenic bone group, with 30 SD rats in each group (half male and half female, female infertile). The time point of test was 2, 4, 12, 26 and 40 weeks, respectively. There were six parallel animals in each time point in each experimental group. After the mice were anesthetized by intraperitoneal injection of sodium pentobarbital (40 mg/ml), the femoral region was sterilized with iodine. The subcutaneous tissue was bluntly separated and the femur was exposed along the two muscle bundles. The periosteum was cut with a surgical knife. A circular hole of 5 mm diameter was drilled near the knee joint at the distal end of the femur to reach the bone marrow cavity. The experimental material was implanted into the hole according to the ratio of 1.2 g/kg body weight [37].

### Roentgenoscopy

The animals were anesthetized at 4, 12 and 40 weeks for X-ray examination. The test parameters are 52 kV, 7.5 mAs, with grids, automatic exposure closure. X-ray film of both hind limbs was taken. Bone repair at bone defect site was observed, such as bone junction, bone healing and bone modeling.

### *Ex vivo* evaluation of the implanted samples

After euthanasia of the experimental animals at 4, 12 and 40 weeks, the femurs were removed and the soft tissue on the femur was removed. The femur was numbered and inserted into the cylindrical foam. The femoral implant was then scanned by conical beam micro-focal X-ray bulb tube micro-CT scanner. The scanning parameters were the resolution size of the scanning was 22 μm; the rotation angle was 360°; the rotation angle increment was 0.4°; 55 kV, 72 μA, 4 weeks.

### Histological observation

Five SD rats were euthanized at 4, 12 and 40 weeks after implantation. The femur was removed and the implanted area was cut off. The femur was washed with PBS three times. All specimens were fixed in 3% formaldehyde solution for 2 weeks, decalcified in 10% EDTA solution and the media was changed every 3 days. After decalcification, dehydration, paraffin embedding, slicing, dyeing and other tissue treatment processes were used to prepare tissue sections. Tissue sections were 5 μm thick and stained with hematoxylin−eosin.

### Immunotoxicity

The test of IgG, IgM was performed as following. The blood of experimental animals was taken by tail vein cut off to test the immune indexes. Peripheral blood of rats was collected in a 1.5 ml centrifugal tube. In order to prepare serum, peripheral blood of rats was centrifuged for 10 min at 1000 g. The upper serum was collected and stored at −20°C. The serum was placed at room temperature for 30 min before use, then tested by ELISA method according to the operation instructions of ELISA kit. At the end of the immune period (4 and 26 weeks), the rats were euthanized, and the spleens were taken aseptically. Each sample was grinded to prepare a single cell suspension. Cell suspension was collected. RPMI1640 containing 10% FCS was added to adjust the cell concentration to 2 × 10^6^ cells/ml. The above lymphocyte suspensions were stained with FITC mouse anti-rat CD4 (Lot: 5299960) and PE mouse anti-rat CD8a (Lot: 6175730) labeled with BioLegend fluorescence, and then analyzed by flow cytometry.

### Compressive test

At 4 weeks, the femurs were dissected and cut into cylinder of 5 mm diameter and 10 mm height by a hard tissue slicer. The precise dimensions of samples were measured by digital calipers. The sample was placed between the two indenters of the test machine and was compressed at a rate of 5 mm/min and compressive strength of each specimen was calculated [38, 39]. The compressive modulus was calculated according to the stress−strain curve.

### Statistical analysis

All data in the experiment were expressed by mean (+SD). The experimental data were compared by AVONA method and *t*-test. The significance level of statistical test was set to *P* < 0.05, **P < 0.01 and ***P < 0.001.

## Results

### Characterization of bone materials

**Figure 1.**
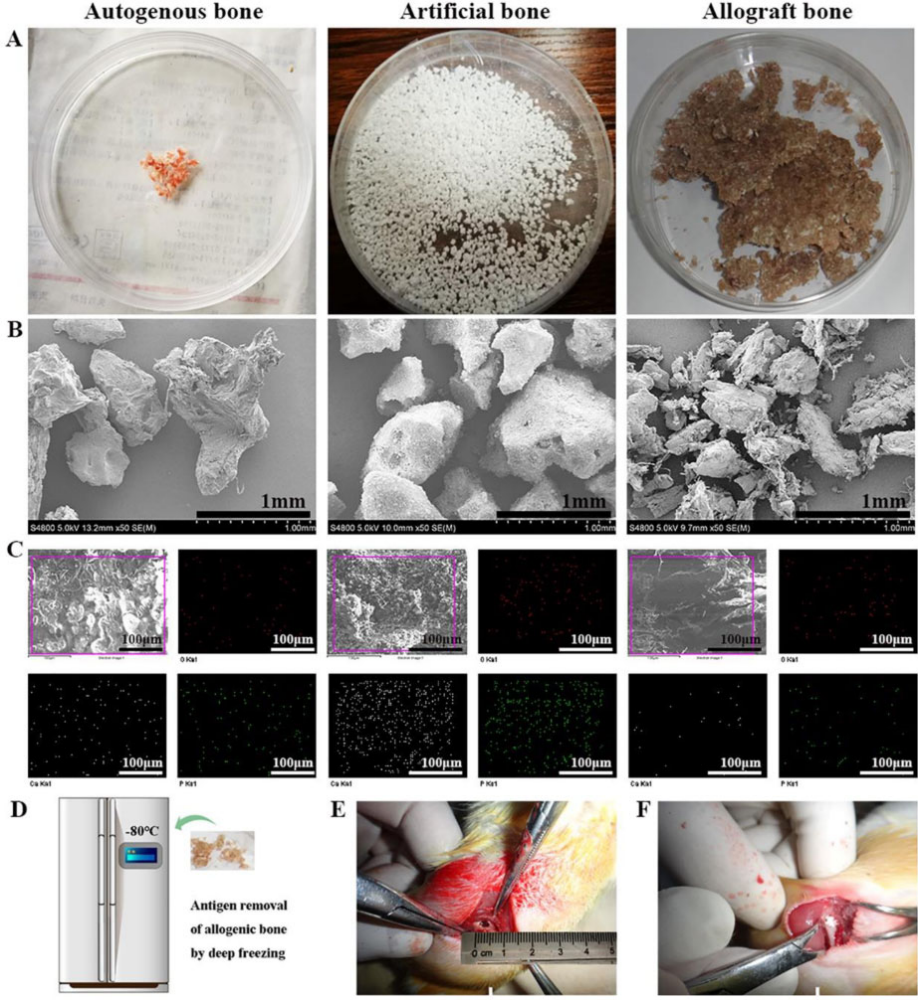
(**A**) Macroscopic images for the implanted materials. (**B**) SEM images of the implanted materials. (**C**) EDS mapping images for the implanted materials. (**D**) Antigen removal by freezing at 80°C. (**E, F**) the implantation process of the materials

The macrograph of autogenous bone, allogenic bone and artificial bone were shown in Fig. 1A. The SEM images of three materials showed the micro-blocks of bone materials (Fig. 1B), the EDS mapping results also showed that there were two elements of Ca and P in three materials (Fig. 1C). Figure 1D illustrated the processes to destroy rat antigens by freezing in a refrigerator at −80°C for 2 weeks for preparation of allogenic bone. Figure 1E and F showed the hole of implantation experiment and the implantation process of the materials. The XRD patterns of autogenous bone and allogenic bone were accordant with standard card of pure HAp (Supplementary Figs S1 and S2). The XRD spectrum of artificial bone substitute material confirmed the biphasic calcium phosphate ceramics of HAp and β-TCP through comparing with standard cards (09-0432, 09-0169) (Supplementary Fig. S3).

### Roentgenoscopy

**Figure 2.**
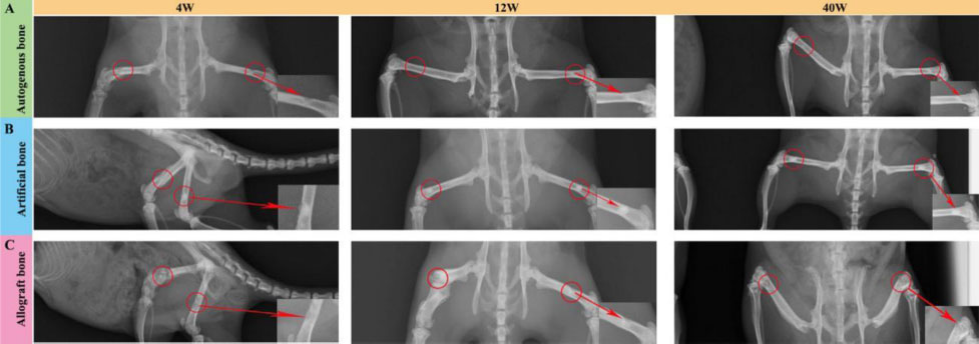
Digital radiography observation of mouse bone defects after implantation with different materials. (**A**) Digital radiography images for autogenous bone group. (**B**) Digital radiography images for artificial bone group. (**C**) Digital radiography images for allograft bone group

Figure 2 shows the typical X-ray images of the surgical site of implantation at different time. At 4 weeks, in autogenous bone group, the bone defect area was intact and homogeneous, and the density of the defect area was significantly lower than that of the surrounding bone tissue. In allogenic bone group, the clear boundary of bone defect area and the low-density shadow was patchy. In artificial bone group, the material boundary between the defect area and the surrounding bone tissue was clear, and the material area showed high density and uniform image. After 12 weeks implantation, the bone defect area was narrowing in autogenous bone group, and autogenous bone material was absorbed while the boundary was blurred. Relatively, unilateral incomplete defect was visual in allogenic bone group, but the middle area showed low-density patch, which was lower than the surrounding normal bone tissue. In artificial bone group, high-density patches, clear boundary between material and surrounding bone tissue were found, and a few callus formations also remained in the defect area. After 40 weeks implantation, in autogenous bone group, the materials in bone defect area were basically absorbed, and some bone defect area was repaired to resemble normal bone tissue, and the medullary cavity was recanalized. The boundary between bone graft and surrounding bone disappeared, and the density of repair area was similar with normal bone. In allogenic bone group, the boundary of femoral defect area was clear and reduced to a certain extent, and flocculent calcification could be seen around the defect. The concave surface was lower than that of the surrounding bone bed. In artificial bone group, most of the femoral defects were repaired, some callus formed and the boundary was blurred.

### Micro-CT

**Figure 3.**
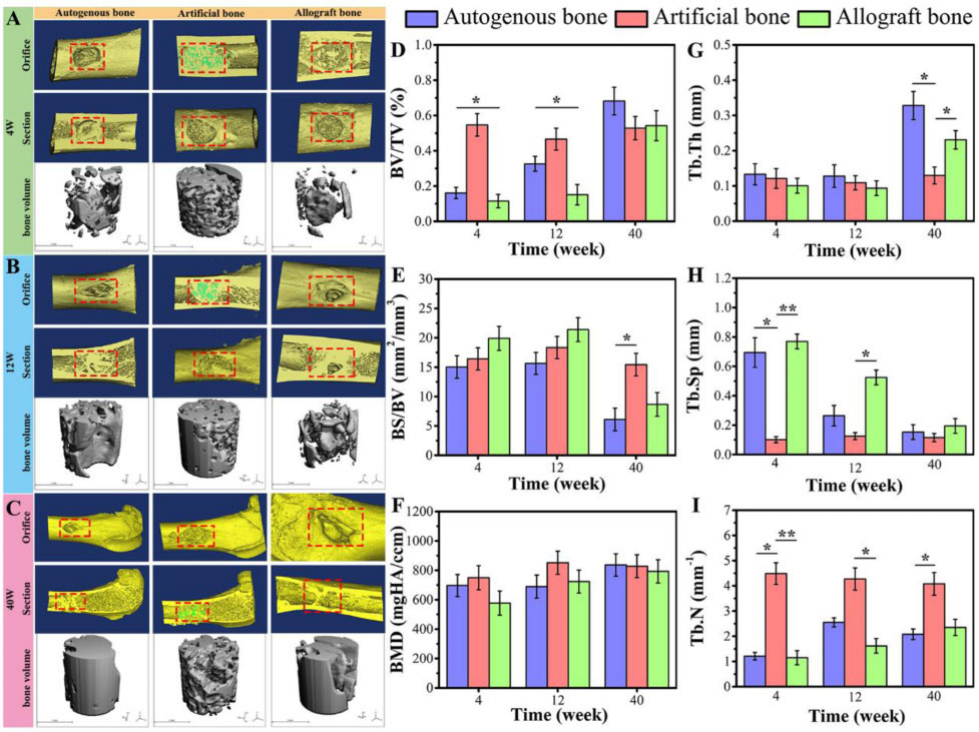
Micro-CT 3D reconstruction analysis for each group of materials during femoral implantation. (**A**) images at 4 weeks; (**B**) images at 12 weeks; (**C**) images at 40 weeks. Implant orifice and longitudinal section were shown in red rectangle. The image of hydroxyapatite was shown in green on the longitudinal section of artificial bone. (**D−I**) Micro-CT parameters of new bone formation inside the graft. Data were presented as mean ± SD by *t*-test (*n* = 3). **P < 0.01; *P < 0.05

The reconstructed 3D models by micro-CT of the three samples were displayed in Fig. 3A−C. In all groups, at 4 weeks, the boundary between the implant materials and the bone tissue around the hole was clear, and the materials were loosely connected with the surrounding bone tissue, indicating that the implanted materials were not connected with surrounding bone tissue. At 12 weeks, there was no obvious boundary between the implanted materials and the bone around the hole, and the materials were connected tightly with the surrounding bone tissue. In autogenous and allogenic bone groups, although the defect holes were narrowed, the holes were not completely repaired. In the artificial bone substitute group, the materials fused closely with the implant hole and the bone defect was repaired well. The above results indicated that osseous connections appeared between the implanted materials and the surrounding bone tissues. After 40 weeks implantation, the materials had better repair effect in all groups. The defective holes were reduced, but not completely repaired to the level of the cortical bone interface. It was worth noting that the surface morphology and characteristics of the orifice in artificial bone group were significantly different from those in autogenous bone and allogenic bone group.

To evaluate bone regeneration inside the grafts, a cylindrical region of interest in each group was chosen in each sample for quantitative analysis. The detailed information on structural parameters was shown in Fig. 3D−I. Bone volume density (bone volume/tissue volume, BV/TV) and bone mineral density in artificial bone group were higher than that in autogenous bone and allogenic bone groups at 4 and 12 weeks. Bone surface density (bone surface area (BS)/BV) showed a downward trend in three groups at 40 weeks, which indicated that the new formed bone gradually grew into the graft at 40 weeks. Moreover, there was no significant difference between three groups in the value of trabecular thickness (Tb.Th) at 4 and 12 weeks; and trabecular number (Tb.N) in artificial bone group was significantly higher than that in autogenous bone and allogenic bone group. The relative low value of trabecular separation (Tb.Sp) in artificial bone group also indicated that artificial bone group had more comprehensive repair performance than other two groups.

### Histological observation

**Figure 4.**
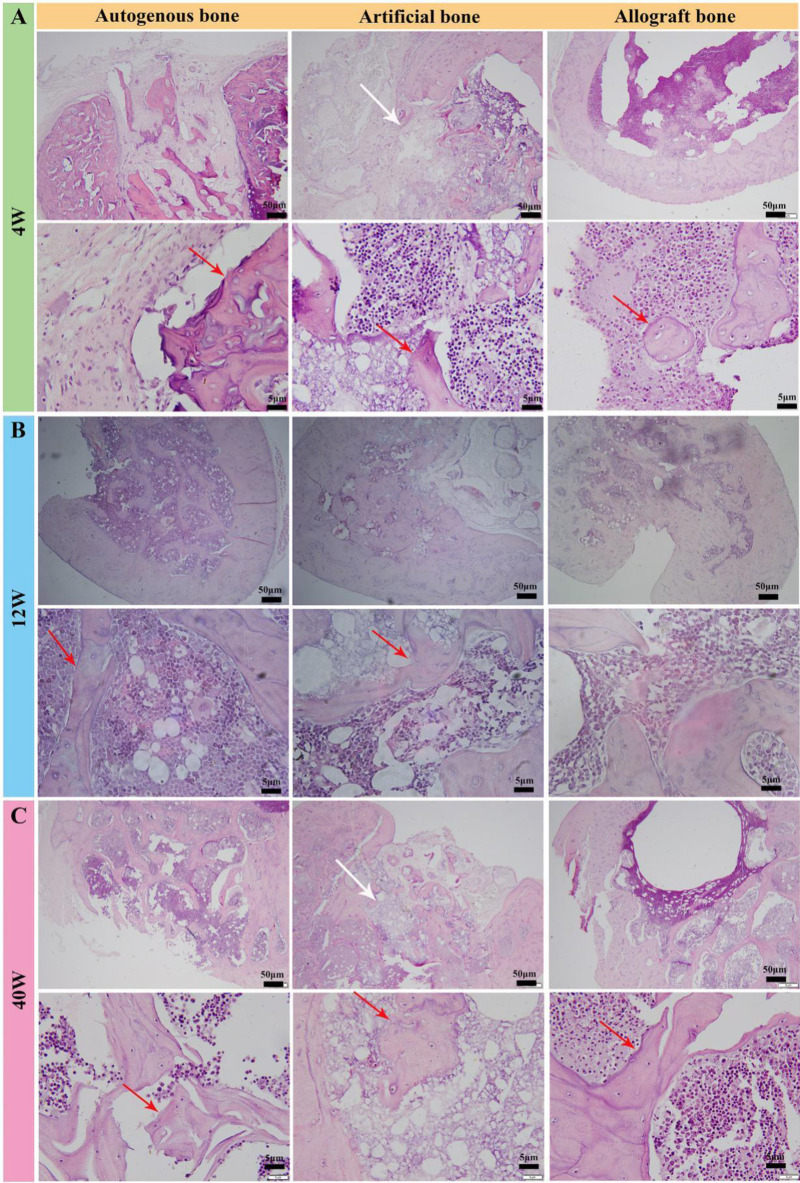
The observation of tissue section after implantation (H&E staining). (**A**) observation of histological sections at 4 weeks. (**B**) Observation of histological sections at 12 weeks. (**C**) Observation of histological sections at 40 weeks

To further detect the bone regeneration throughout the three bone grafts, cross-sections of the grafts were assessed by histological observation. As shown in Fig. 4A, new red light stained trabecular bone tissue could be seen in the orifice of bone implants in autogenous bone group at 4 weeks after implantation. Bone cells and bone lacunae were found in the trabecular bone tissue under high magnification microscopy. Gray fibrous connective tissue could also be seen beside the trabecular bone. Relatively, fragments of bone tissue clustered in the defect position filled with allogenic bone graft, and a large number of cells surrounded the allogenic bone substitute material. At high magnification, apparently active bone tissue was found, indicating that the graft began to live and grow. In artificial bone group, gray artificial bone material after decalcification could be seen in the orifices of bone implants (white arrow), and a small amount of red light stained bone trabecular tissue appeared in the implants. But the visual content of new bone tissue was less than that of autogenous bone tissue.

At 12 weeks, reticulated bone trabeculae in the implantation orifice and medullary cavity was observed in autogenous bone group, some of which had been calcified into bone tissue, and a large number of bone marrow cells around the bone tissue were seen under high power microscopy (Fig. 4B). Similarly, the newly formed trabeculae could be observed after implantation of artificial and allogenic bone grafts for 12 weeks. However, it showed a significantly weaker filling effect than autogenous bone graft due to the larger defect area (yellow). In Fig. 4C, a large number of reticulated bone tissues in the orifice and medullary cavity were presented in autogenous bone implantation group, and showed obvious mineralization and osteogenesis. Although the allogenic and artificial bone groups also showed good osteogenic effect, the visual repair process was significantly weaker than that of autogenous bone group. These results were consistent with the analysis of CT data in Fig. 3.

### The compression test of three bone grafts

**Figure 5.**
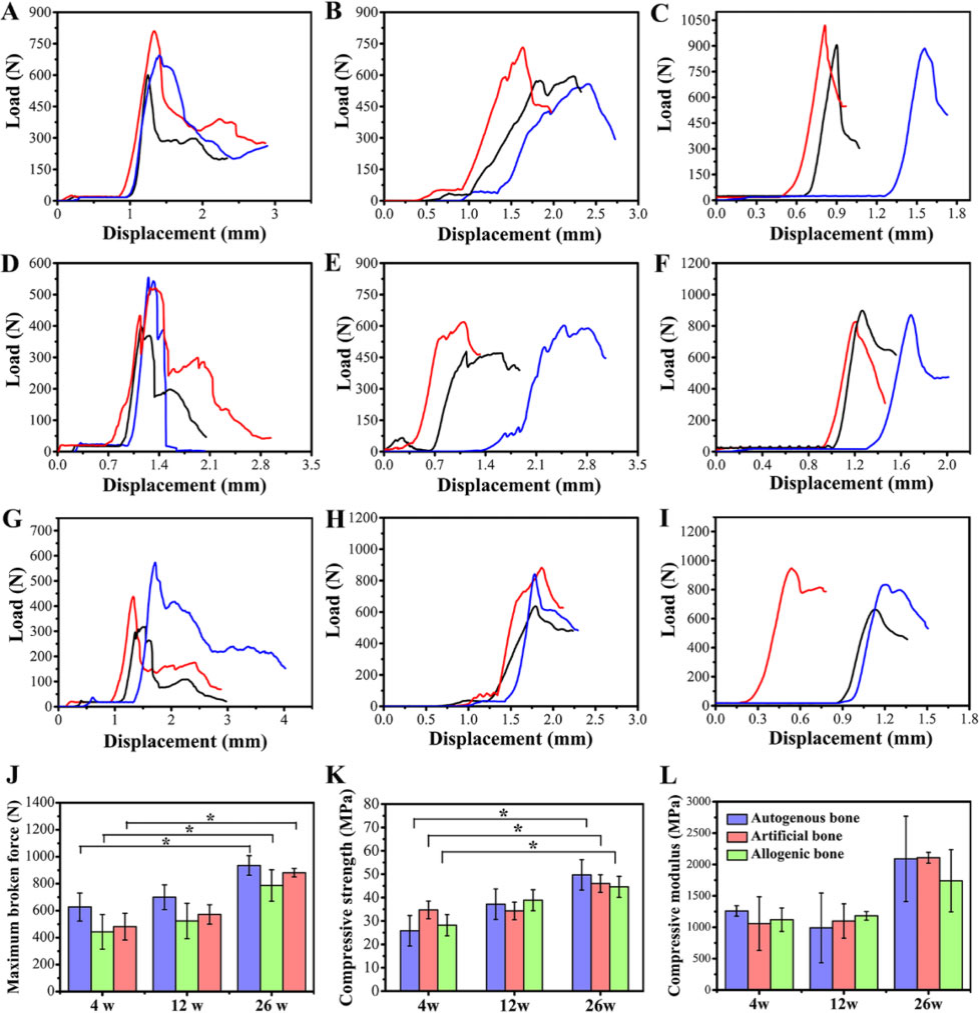
Compression test of femur in animals of different groups. (**A−C**) The change of load with displacement of autogenous bone at 4, 12 and 26 weeks. (**D−F**) The change of load with displacement of artificial bone at 4, 12 and 26 weeks. (**G−I**) The change of load with displacement of allograft bone at 4, 12 and 26 weeks. (**J**) Maximum broken force derived from the curve of compression test. (**K**) Statistical analysis of changes in femoral compressive strength of animals in each group. (**L**) Statistical analysis of changes in femoral compressive modulus of animals in each group. Data were presented as mean ± SD by *t*-test (*n* = 3). *P < 0.05

The compression test results of different implant samples at different implantation time points were shown in Fig. 5. The change of load with displacement of samples was shown in Fig. 5A−I, and the maximum broken force derived from the curve of compression test was shown in Fig. 5J. Due to the voids between the indenter and the sample, a horizontal line appeared at the beginning of the load−displacement curves. The load increased after the indenter attached the sample. The maximum broken force of three grafts increased with the elongation of implantation time, and the maximum broken force in artificial bone group was closer to that in autogenous bone group compared with that in allogenic bone group. The stress−strain curves of three grafts at three different time points were shown in Supplementary Fig. S4, and the compressive strength of three grafts was shown in Fig. 5K. The compressive strengths of autogenous bone group, artificial bone group and allogenic bone group at 4 weeks were 25.83, 34.75 and 28.2 MPa, respectively. At 12 weeks, the compressive strengths of autogenous bone group, artificial bone group and allogenic bone group were 37.18, 34.31 and 38.88 MPa, respectively. After 26 weeks implanted, the compressive strengths were 49.7, 46.0 and 44.6 MPa, respectively. There was no significant difference in compressive strength and maximum broken force of autogenous bone between 4 and 12 weeks or 12 and 26 weeks. However, there was a significant difference between 4 and 26 weeks. The results showed that the compressive strength was significantly strengthened at 26 weeks, implying the strong repairing ability of autogenous bone. The compressive modulus in Fig. 5L showed similar tendency with the strength data (Fig. 5K). Meanwhile, the micro-CT results showed that the bone surface density of the three groups did not show significant difference at the early stage of implantation, suggesting that all three materials were beneficial to bone regeneration and repair after prolonged implantation. After long-term implantation, in autogenous and allogenic bone groups, but the artificial bone group did not show significant difference between 4, 12 and 40 weeks (Fig. 3E). After long-term implantation, bone surface density in autogenous and allogenic bone groups decreased, but that of artificial bone did not decrease significantly. This may mainly be ascribed to the relatively slow degradation of biphasic calcium phosphate ceramics [40].

### Immunotoxicity test of three bone grafts

**Figure 6.**
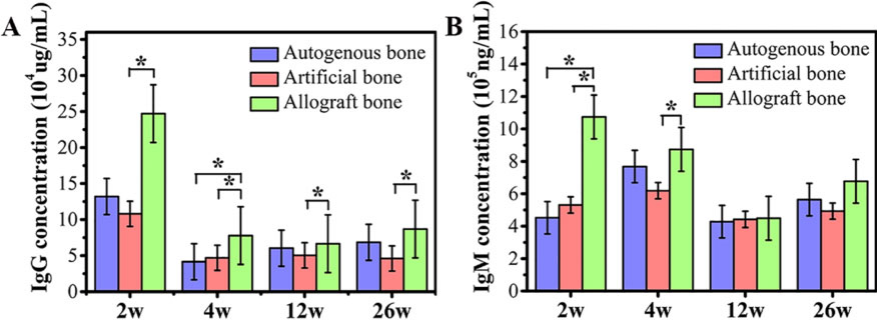
Immunoglobulin content analysis. (**A**) IgG content; (**B**) IgM content. Data were presented as mean ± SD by *t*-test (*n* = 6). *P < 0.05

The change of antibody concentration was the main manifestation of immune response. IgG, IgM and other antibodies were immunoglobulins that could specifically be combined with antigens and played an important role in the immune response of biomaterials [41]. As shown in Fig. 6A, the results of IgG detection showed that there was no significant difference in the concentration of IgG between artificial bone group and autogenous bone group. But the IgG of artificial bone group was significantly lower than that of allogenic bone group. Similarly, after 2 weeks of immunization, the concentration of IgM in artificial bone group and autogenous bone group were significantly lower than that in allogenic bone group, but no significant difference between artificial bone group and autogenous bone group was observed (Fig. 6B). At the end of the 4 weeks immunization, significant differences remained between allogenic bone group and artificial bone group. But there was no significant difference in serum IgM levels among the three groups after 12- and 26-week immunization.

**Figure 7.**
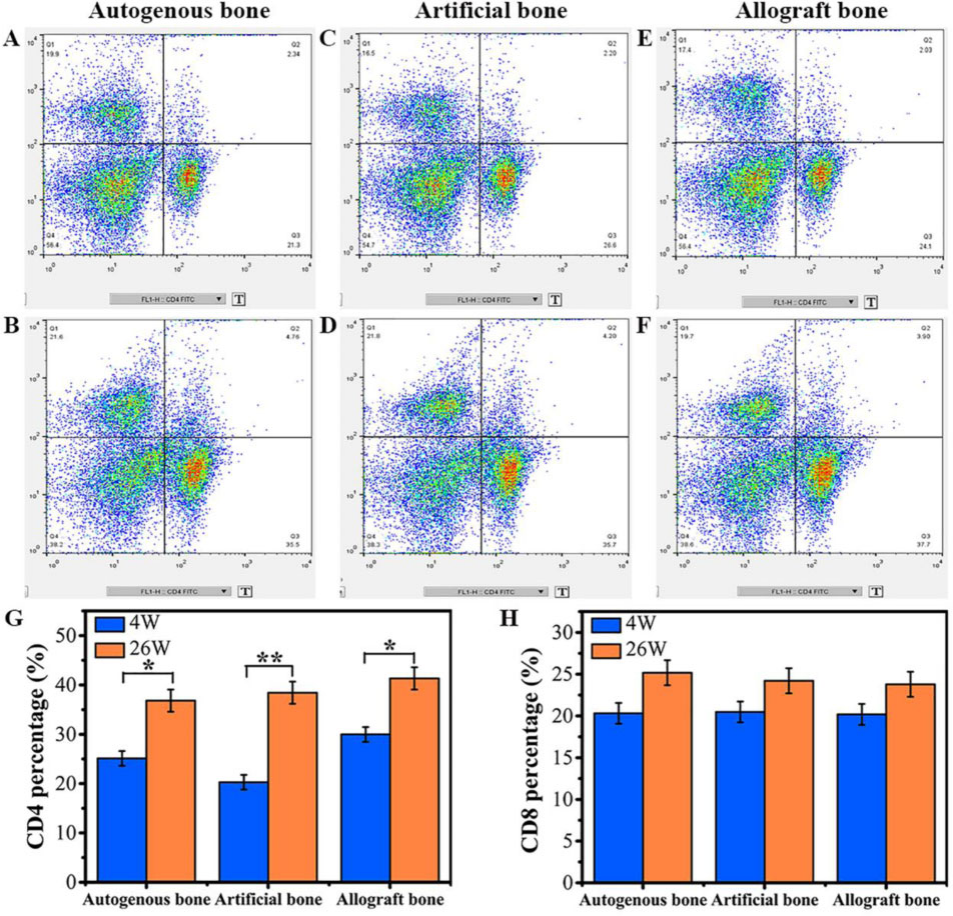
CD4 and CD8 lymphocyte typing. (**A, B**) Lymphocyte typing for autogenous bone at 4 and 26 weeks. (**C, D**) Lymphocyte typing for artificial bone at 4 and 26 weeks. (**E, F**) Lymphocyte typing for allogenic bone at 4 and 26 weeks. (**G, H**) Contents of CD4 and CD8 at 4 and 26 weeks. Data were presented as mean ± SD by *t*-test (*n* = 5). **P < 0.01; *P < 0.05

The percentage of CD4 and CD8 lymphocyte expression was one of the important indicators of immune system function, especially the immune regulation function of organism. Flow cytometry results of the three groups indicated that the CD4 and CD8 expression of spleen lymphocytes in three groups increased with time extension (Fig. 7A−F). The quantitative analysis of CD4 and CD8 was exhibited in Fig. 7G and H. At 4 weeks, there was no significant difference in CD8 percentage of spleen lymphocyte among three groups, but obvious enhanced CD4 expression was found in allogenic bone group. As the implantation time increased to 26 weeks, no significant difference was presented in CD4 and CD8 percentage of spleen lymphocyte.

## Discussion

The main purpose of clinical bone defect repair is to reconstruct the bone structure and restore the physiological function of bone. In this study, a rat femur defect model was established. Autogenous, allograft and artificial bone substitutes were implanted in the defect sites; their bone regeneration and immunotoxicity were investigated. The X-ray, gross observation, micro-CT, histological observation and mechanical property results showed that, after implanting *in vivo* for a period of time, all these materials promoted the formation of new bone. Among them, autogenous bone and artificial bone substitutes had similar strong ability to repair bone defects. However, the autogenous bone was taken from the iliac bone of experimental animals. It had been reported that the increased incision, prolonged operation and anesthesia time damaged bone structure and functional stability of donor site, and might result in donor site complications [42, 43]. In addition, available amount of autogenous bone was limited. Artificial bone substitutes with good biocompatibility and excellent bone induction capability could promote osteogenesis and new bone formation [44–47]. Hence, it provided a good choice for bone defect repair in clinic.

On the other hand, allogeneic bone grafts exhibited worse repairing ability compared with other two materials. Besides, higher level concentration of immunoglobulins (IgG, IgM) was found in allogeneic bone group, which was consistent with the results of histological observation. The relatively worse repairing ability of allogenic bone was probably due to the immunotoxicity of the material. Because of the relationship between immune rejection and immunotoxicology, the main problems in clinical practice of allogenic bone were delayed healing of bone, unsatisfactory vascularization, low healing strength and non-union [48–50]. The immunotoxicity of materials was related to the characteristics of materials. There were many allergic substances such as animal protein, polypeptide, polysaccharide and other macromolecular substances. They had both immunogenicity and reactivity. In complete antigen, it could directly stimulate the immune system to produce immune response, made the body producing antibodies or sensitized lymphocyte, and finally lead to allergic reaction [41, 51]. Next, the micro−nano structure of materials could change the host antigens by interacted with various histones *in vivo*, leading to cell loss or cell death [52–54]. The transformation of hapten into whole antigen caused immune system reaction and led to immunotoxicity. Artificial bone had strong protein adsorption ability, which might cause immunotoxicity after protein adsorption *in vivo* [5, 11, 20, 25]. However, the results of this study in Fig. 6A and B show that no increase of IgG and IgM concentration were observed in the artificial bone, which indicated that the antigen concentration formed by protein adsorption in the artificial bone was low.

The analysis of CD4 and CD8 positive expression of lymphocyte in spleen and other immune organs by flow cytometry was used to explore whether medical devices contacted with organism had potential ability to affect the immune system function of organism [55, 56]. At 4 weeks, the percentage of CD4 cells in allogenic bone group was higher than that of artificial bone and autogenous bone group, which indicated that CD4 T cells were involved in the immune rejection of allogenic bone. There was no significant difference between the percentage of CD8 T cells in autologous bone, artificial bone and allogenic bone, indicating that CD8 T cells had not been involved in immune rejection. Therefore, it is reasonable to believe that the immunogenicity of 3 bone substitutes was moderate and acceptable as bone implanting materials [57]. However, at different stages *in vivo*, especially at the early stage of implantation, the immunological indexes such as IgG, IgM concentration and CD4 T cells population of allogenic bone significantly increased to the degrees that were significantly higher than that of autogenous/artificial bone. Even after deep freezing, the antigenicity of allogenic bone still remained difficult to totally eliminate [58]. These results implied that allogenic bone might stimulate higher humoral immune responses than that of autogenous bone and artificial bone, which might be influence the regeneration of neo-bone tissue. On the other hand, the artificial bone could not only induce bone formation but also rarely stimulate the antigen-antibody reaction *in vivo* and did not produce humoral immune toxicity reaction [46, 59].

## Conclusions

In this study, the osteogenesis effect of autogenous, allogenic and biphasic calcium phosphate artificial bone substitutes on rat femur defect model were investigated *in vivo*. The results showed that artificial bone substitute, which contained 61.4% HA and 38.6% β-tricalcium phosphate with 61.64% porosity and 0.8617 ± 0.0068 g/cm^3^ density (*d* ≤ 2mm), had strong bone repair ability, similar with that of autogenous bone graft. On the other hand, the bone repair ability of allogenic bone graft was relatively poor, even after antigen removal by freeze−thawing treatment, probably due to immunotoxicological reaction of materials. This study provided experimental basis for the selection of bone repair materials for clinical use. Although the repair process of artificial bone was relatively inefficient than autologous bone graft, the low immunotoxicological indexes and acceptable therapeutic effects endowed it an excellent alternative material to solve the problems of insufficient source and secondary trauma of autogenous bone.

## Supplementary data

Supplementary data are available at *REGBIO* online.

## Funding

This work was supported by National Key Research and Development Program of China (2018YFC1106800) and Sichuan province key research and development project (20ZDYF0191). The authors greatly appreciated Cheng Li from Analytical & Testing Center of Sichuan University for her assistance in Micro-CT measurement. 

*Conflict of interest statement*. The authors declare no competing non-financial/ﬁnancial interest. 

## Supplementary Material

rbaa040_Supplementary_Data
