# Supplementary material for: A comparative study of autogenous, allograft and artificial bone substitutes on bone regeneration and immunotoxicity in rat femur defect model
Source: Regen Biomater. 2020 Sep 30;8(1):rbaa040. doi: 10.1093/rb/rbaa040 (PMC7947581; doi:10.1093/rb/rbaa040)
Supplement: rbaa040_Supplementary_Data [file rbaa040_supplementary_data.zip › Revised support information.docx]

**A comparative study of** **autogenous, allograft, and artificial bone substitutes on bone regeneration and immunotoxicity i****n rat femur defect model**

Wen Zou^1,2^, Xing Li^1^, Na Li^2^, Tianwei Guo^2^, Yongfu Cai^2^, Xiaoqin Yang^2^, Jie Liang^1,2*^, Yong Sun^1^, Yujiang Fan^1^

^1^National Engineering Research Center for Biomaterials, Sichuan University, Chengdu 610064, Sichuan, china.

^2^Sichuan Testing Centre for Biomaterials and Medical devices, Chengdu 610064, Sichuan, china.

**Author information**

**Corresponding Author**

*Prof. Jie Liang

Tel: +86-28-85416196 Fax: +86-28-85416196

E-mail: [jie_L88@126.com](mailto:jie_L88@126.com)

**
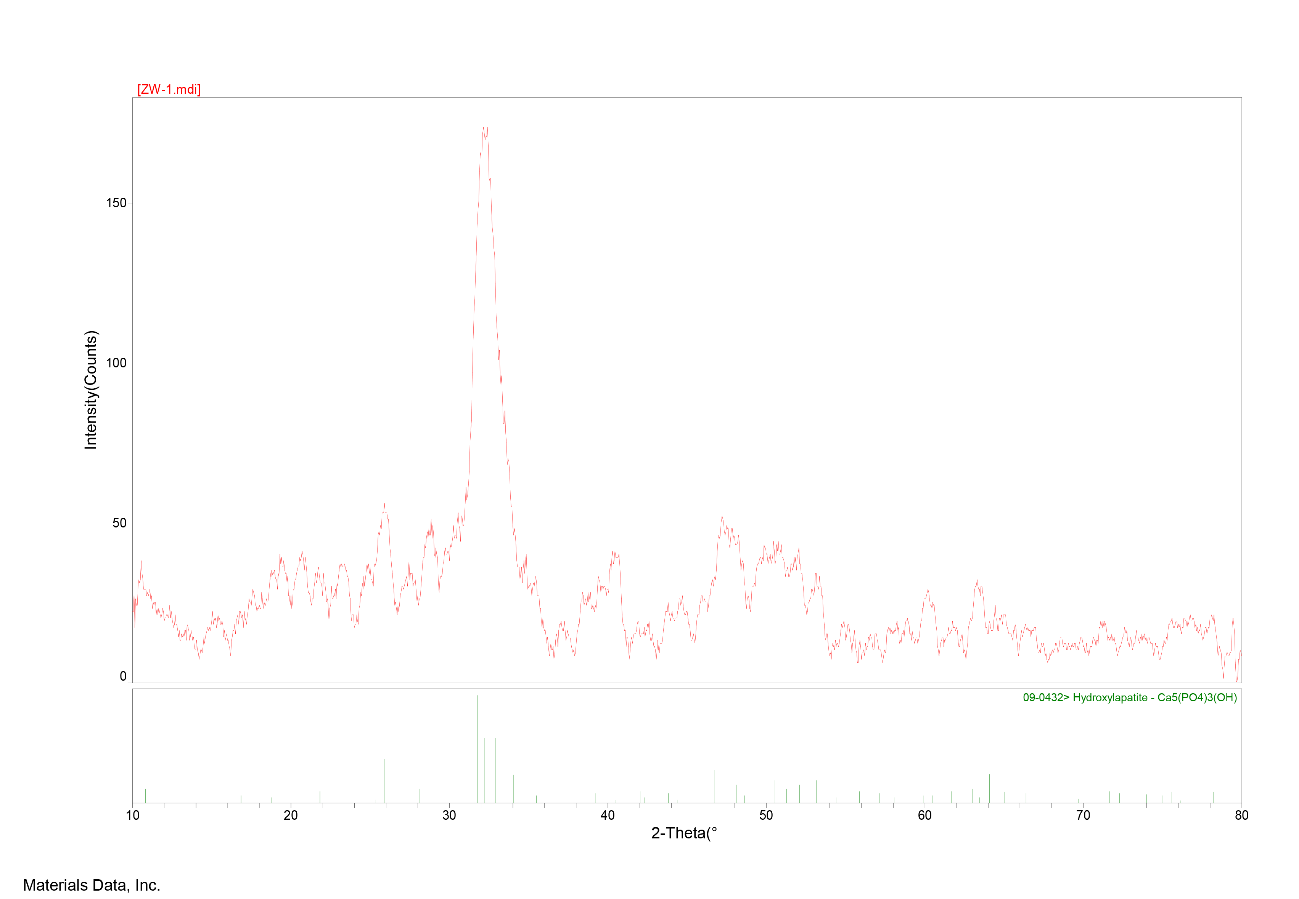
**

Figure S1. XRD pattern of autogenous bone.

**
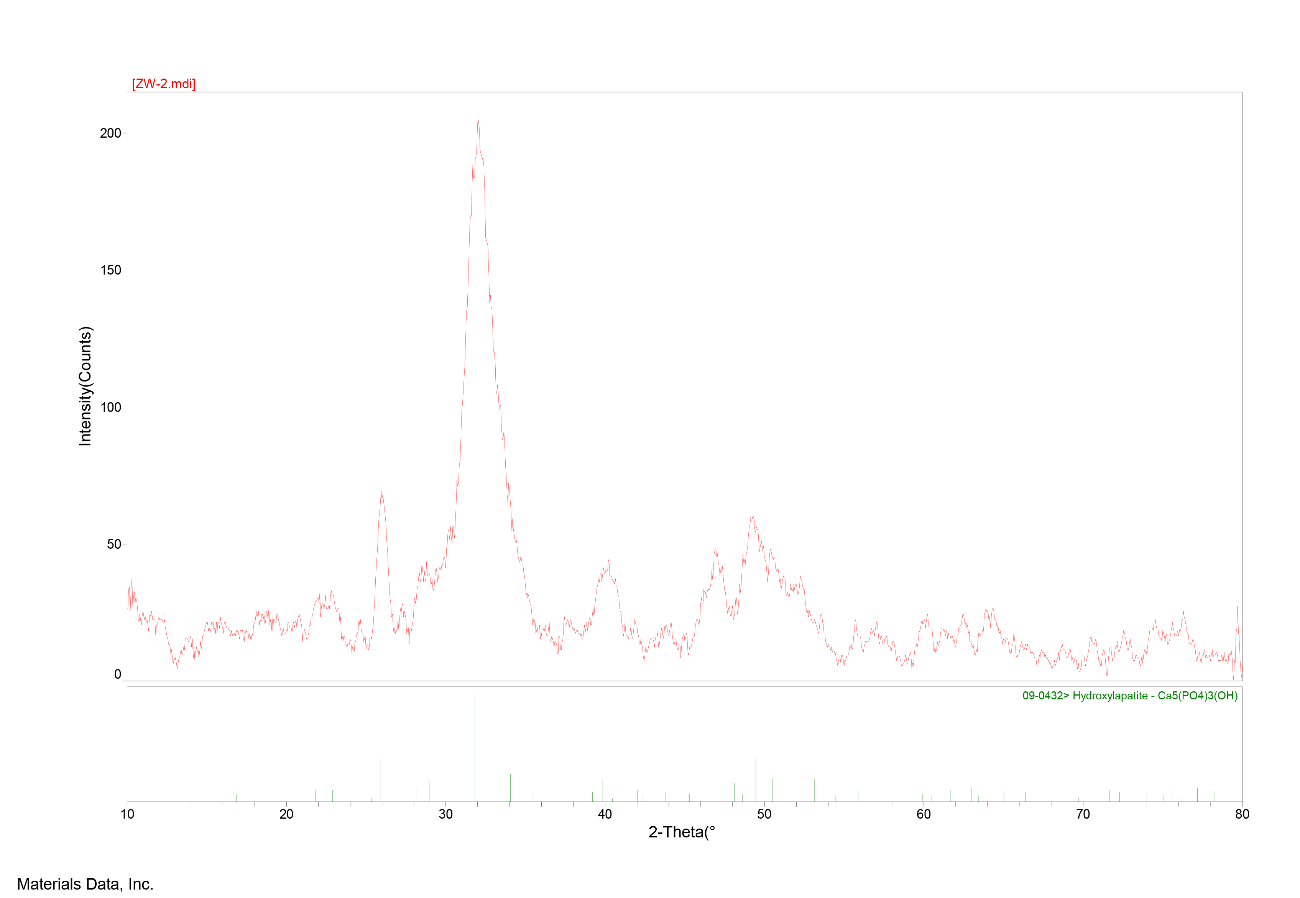
**

Figure S2. XRD pattern of allograft bone.


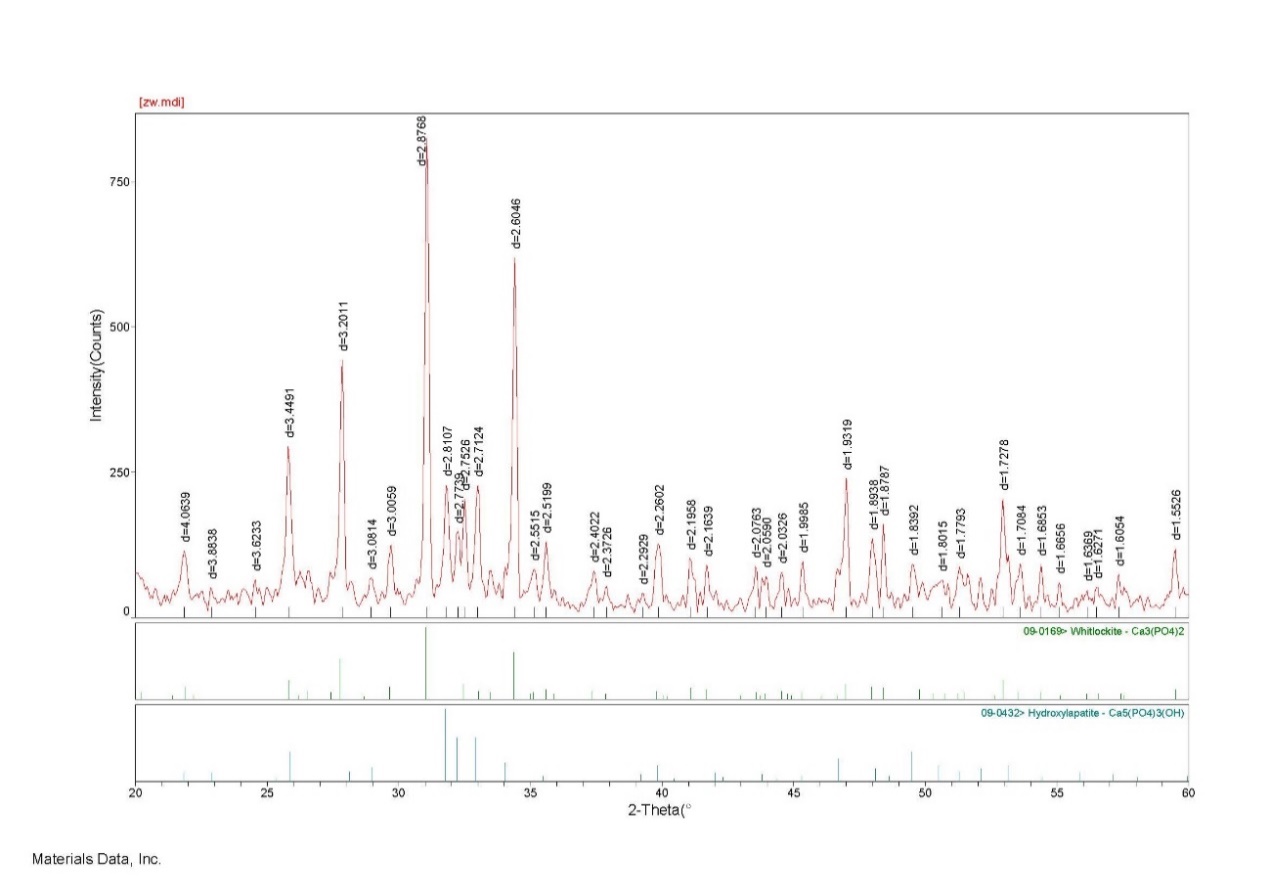


Figure S3. XRD pattern of artificial bone.


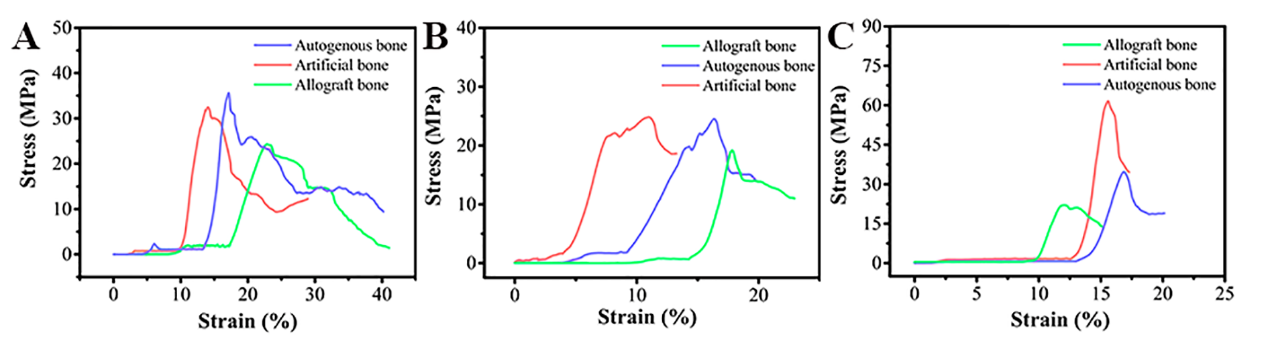


Figure S4. Stress-Strain curves of three grafts after 4 weeks (A) 12 weeks (B) 26 weeks (C) implantation
