# Supplementary material for: A comparative study of autogenous, allograft and artificial bone substitutes on bone regeneration and immunotoxicity in rat femur defect model
Source: Regen Biomater. 2020 Sep 30;8(1):rbaa040. doi: 10.1093/rb/rbaa040 (PMC7947581; doi:10.1093/rb/rbaa040)
Supplement: rbaa040_Supplementary_Data [file rbaa040_supplementary_data.zip › Response to Referee.docx]

Dear Dr. Peppas,

Thank you very much for your attention on our manuscript.

We highly appreciate the valuable comments and suggestions of the reviewers, which greatly helped us to improve the quality of our manuscript. According to these comments and suggestions, we revised the manuscript carefully. All the changes in the revised manuscript was marked in yellow for the convenience of the reviewers.

We hereby submit our revised manuscript entitled “A comparative study of autogenous bone, allograft bone, and artificial bone substitutes on bone regeneration and immunotoxicity in rat femur defect model” for your consideration. The point-to-point responses to the reviewers’ comments were listed as follow:

**The responses to Reviewer #1:**

Comments to the Author

The manuscript evaluated the bone defect repairing ability of autogenous bone, allogenic bone and artificial bone in many aspects. The mechanical properties, micro-CT imaging analysis, digital image analysis and histological slice analysis and immune reaction of the three bone implants were systematically investigated. All experiments were well performed. However, the manuscript was not sufficiently sound for publication in the journal unless a minor revision is performed. The following suggestions should be considered:

*1. In abstract section, the author mentioned that autogenous bone implant materials caused greater secondary damage to experimental animals. However, the author did not discuss the damage effect on experimental animals later.*

**ANSWER:** Accordingly, related description was added in the discussion part in the revised manuscript on Page 12-13.

*2. The EDS mapping images in Fig.1 c were not matched with the SEM images. In addition, please added scale-bar in SEM images and EDS mapping images.*

**ANSWER:** Thank you very much for your valuable suggestions. The corresponding SEM images and scale-bar in SEM images and EDS mapping images has been added in new Fig 1.

*3. Modulus as an important parameter for bone implants. Please provide the compressive modulus data. Some current researches regarding mechanical requirements of bone implants may provide some guidelines during designing.*

*a. A strawberry-like Ag-decorated barium titanate enhances piezoelectric and antibacterial activities of polymer scaffold. Nano Energy, 2020, 74: 104825.*

*b.* *A graphene oxide-Ag co-dispersing nanosystem: dual synergistic effects on antibacterial activities and mechanical properties of polymer scaffolds. Chemical Engineering Journal, 2018, 324:322-333.*

**ANSWER:** The compressive modulus data was added in the Results part on page 11 of the revised manuscript. Accordingly, the methods for compressive modulus measurement from the two references were cited in Experimental part on Page 7 of the revised manuscript. The corresponding Stress-Strain curves of three bone grafts were exhibited in Figure S4.

*4. Please explain the horizontal line at the beginning of load-displacement curves. In addition, please discuss the linkage between bone surface density and compressive strength.*

**ANSWER:** In the process of gradual compression, the testing indenter does not touch the sample, but the indenter moves at a constant speed. At this time, the pressure is 0, so a horizontal line at the beginning is generated. In addition, bone surface density and mechanical properties were also discussed. We added a description about this phenomenon on Page 11.

*5. In discussion section, the author only focused on the immune reaction of the bone implants. Please comprehensive discuss each properties and demonstration the inner linkage among them.*

**ANSWER:** According to the suggestion, more detailed discussion about each properties and inner linkage among them has been added in the Discussion part of the revised manuscript.

*6. Some state-of-art researches investigation regarding biomaterials applied in artificial bone should be included:*

*a. Metal organic frameworks as a compatible reinforcement in a biopolymer bone scaffold. Materials Chemistry Frontiers 4.3 (2020): 973-984.*

*b.* *Molybdenum disulfidenanosheets embedded with nanodiamond particles: co-dispersion nanostructures as reinforcements for polymer scaffolds. Applied Materials Today, 2019, 17: 216-226.*

**ANSWER:** According to the suggestion, relevant description and citation have been added in Introduction part as reference [29] and [30], respectively, on Page 4.

**The responses to Reviewer #2.**

Comments to the Author

In this work, the authors compared the bone regeneration effect and immuno-toxicity of 3 bone substitute materials. The employed a rat bone defect model to evaluate the mechanical properties, micro-CT images, digital images, histology the 3 materials after different time points of implantation. By comparing the results, they found that artificial bone substituent had similar osteogenic capacity with autogenous bone substitute. On the other hand, allogenic bone exhibited poor bone defect repair ability. Further, they found that compared with artificial and autogenous bone substitutes, the early stage immunological indexes such as IgG, IgM concentration and CD4 cells population of allogenic bone were significantly higher, implying that the immunotoxicity affected the bone repair ability of allogenic bone substitute. These results might be meaningful for seeking the proper filling materials for bone defect regeneration. I would like to recommend the publication of this work after the following minor revision.

*1. The authors should provide more detailed information about the calcium phosphate ceramic particles, including the phase composition, porosity, density, etc, since these parameters would greatly influence the bone formation of bioceramics.*

**ANSWER:** According to the suggestion, detailed information about the calcium phosphate ceramic particles such the phase composition, porosity and density has been added in the Material and Methods part on Page 4 of the revised manuscript and has been highlighted by yellow.

*2. The description of the samples for compressive tests was missing. Please indicate the size, cutting methods, etc.*

**ANSWER:** According to the suggestion, corresponding detailed description has been added in the Material and Methods part on Page 7 of the revised manuscript and has been highlighted by yellow.

*3.* *CD 4 percentages were significantly increased after 26 w, comparing with 4 w, but CD 8 percentages did not change significantly. Here, more detailed discussion was necessary about this phenomenon, in referencing the literatures on the functions of CD4 cells and CD8 cells in the bone regeneration processes.*

**ANSWER:** Actually, the immune responses are directly related with the regeneration processes. CD 8 cells, as the cytotoxic T cells, identify antigen substances that bind to the surface of other immune cells. Limited percentage of CD8 cells implied that the immunogenicity of the materials was moderate, suggesting that these materials were acceptable as bone substitute materials, as manifested in the wide application of these 3 materials in clinic practice. However, the increased CD4 percentage and IgG, IgM in allogenic bone indicated that it might stimulate the humoral immune responses, which might be the originate for the relatively low bone forming capability of allogenic bone, comparing with autogenous and artificial bone. Accordingly, discussions were added after referencing some relevant literatures in the Discussion part of the revised manuscript on Page 14.

*4. Please check the linguistic accuracy to prevent typo and grammar errors.*

**ANSWER:** According to the suggestion, we revised the manuscript carefully.

**Review Editor**

Comments to the Author:

An important topic closely related to regenerative biomaterials!

Based on the comments of the two referees and also my own reading of manuscript, I would like to suggest acceptance of this manuscript after minor revision.

Besides the comments addressed by the two referees, I have the following suggestions:

*1. The title “A comparative study of autogenous bone, allograft bone, and artificial bone substitutes on bone regeneration and immunotoxicity” is overly general. The animal model should be indicated in the title.*

**ANSWER:** According to the suggestion, the title was revised as “A comparative study of autogenous, allograft, and artificial bone substitutes on bone regeneration and immunotoxicity in rat femur defect model” to specify the animal model.

*2. The animal model should also be indicated in abstract. Additionally, the biomaterial of “artificial bone substitutes” should be* *explicitly indicated in ABSTRATC.*

**ANSWER:** According to the suggestion, the rat animal model has been mentioned in abstract, and the description of “artificial bone substitutes” was also added in abstract on Page 2.

*3. Page 4, line 18, delete “It”.*

**ANSWER: We apologize this mistake.** The redundant “It” located at the end of Line 8 of Page 4 in the previous version. This typo was corrected.

*4. Discussion could be presented in more details. Some pertinent publications could be mentioned, including but not limited to, Regenerative Biomaterials, 2018, 43–59; Regenerative Biomaterials, 2018, 129–139; Regenerative Biomaterials, 2018, 115–124; Regenerative Biomaterials, 2018, 85–92; Regenerative Biomaterials, 2018, 125–128; Int. J. Nanomed., 8: 1855-1865 (2013); Regen. Biomater., 2(1), 9-19 (2015); Regenerative Biomaterials, 2020, 303–311; Regenerative Biomaterials, 2020, 233–245; Regenerative Biomaterials, 2020, 71–75; Regenerative Biomaterials, 2020, 71–75; Regenerative Biomaterials, 2019, 29–37; Regenerative Biomaterials, 2019, 349–359; Regenerative Biomaterials, 2019, 335–347;*

**ANSWER:** Thank you very much for your valuable suggestions. More detailed discussion about the comparation of bone formation between the autogenous, allogenic and artificial bone substitutes has been added in the revised manuscript in the beginning of the Discussion part with the citation of related references.

*5. Again, the animal model should be indicated in Conclusion, and the conclusion is suggested to be restricted in a range instead of two general. Besides chemistry, the geometry feathers of the “artificial bone substitutes” could also be described. The biomaterial could This is an original article instead of a review.*

**ANSWER:** Thank you very much for your valuable suggestions. The animal model, as well as the chemistry and geometry information about artificial bone substitutes has been added on Page 14.

We look forward to hearing from you.

Thanks and all the best.

Yours sincerely,

Prof. Jie Liang

National Engineering Research Center for Biomaterials

Sichuan University

Chengdu, China
